# First In-Human Medical Imaging with a PASylated ^89^Zr-Labeled Anti-HER2 Fab-Fragment in a Patient with Metastatic Breast Cancer

**DOI:** 10.1007/s13139-020-00638-7

**Published:** 2020-04-20

**Authors:** Antonia Richter, Karina Knorr, Martin Schlapschy, Stephanie Robu, Volker Morath, Claudia Mendler, Hsi-Yu Yen, Katja Steiger, Marion Kiechle, Wolfgang Weber, Arne Skerra, Markus Schwaiger

**Affiliations:** 1 Nuklearmedizinische Klinik und Poliklinik, Klinikum rechts der Isar, Technische Universität München, Ismaninger Straße 22, 81675 Munich, Germany; 2 Lehrstuhl für Biologische Chemie, Technische Universität München, Emil-Erlenmeyer-Forum 5, 85354 Freising, Germany; 3 Comparative Experimental Pathology, Institut für Allgemeine Pathologie und Pathologische Anatomie, Technische Universität München, 81675 Munich, Germany; 4 Department of Gynaecology, Klinikum rechts der Isar, Technische Universität München, 81675 Munich, Germany; 5 Klinikum rechts der Isar, Technische Universität München, 81675 Munich, Germany

**Keywords:** Breast cancer (BCa), Fab fragment, Human epidermal growth factor receptor 2 (HER2), Imaging, PASylation, ^89^Zr

## Abstract

**Purpose:**

PASylation® offers the ability to systematically tune and optimize the pharmacokinetics of protein tracers for molecular imaging. Here we report the first clinical translation of a PASylated Fab fragment (^89^Zr∙Df-HER2-Fab-PAS_200_) for the molecular imaging of tumor-related HER2 expression.

**Methods:**

A patient with HER2-positive metastatic breast cancer received 37 MBq of ^89^Zr∙Df-HER2-Fab-PAS_200_ at a total mass dose of 70 μg. PET/CT was carried out 6, 24, and 45 h after injection, followed by image analysis of biodistribution, normal organ uptake, and lesion targeting.

**Results:**

Images show a biodistribution typical for protein tracers, characterized by a prominent blood pool 6 h p.i., which decreased over time. Lesions were detectable as early as 24 h p.i. ^89^Zr∙Df-HER2-Fab-PAS_200_ was tolerated well.

**Conclusion:**

This study demonstrates that a PASylated Fab tracer shows appropriate blood clearance to allow sensitive visualization of small tumor lesions in a clinical setting.

## Introduction

Human epidermal growth factor receptor 2 (HER2) is a cell membrane receptor tyrosine kinase that plays a key role in cell development, proliferation, and differentiation [1]. Moreover, HER2 is overexpressed in a variety of cancers, including bladder; lung; gastric; ovarian; prostate; and, in particular, breast cancer (BCa) [2]. Overexpression of HER2 on tumor cells is associated with a high rate of proliferation and aggressive disease, poor prognosis, and short overall survival [3]. Notably, HER2-targeted therapies with monoclonal antibodies (mAbs) such as trastuzumab (Herceptin; Genentech, South San Francisco, CA) have significantly improved survival for up to 20% of patients suffering from BCa [4].

Precise determination of HER2 expression is the basis for success of HER2-targeted therapy. Routinely, HER2 status in BCa is determined on tissue biopsies either via immunohistochemistry (IHC) or by fluorescence in situ hybridization (FISH). However, both methods may be inaccurate in up to 20% of cases [5]. Also, due to the small size and limited number of tissue samples, tumor heterogeneity poses a challenge [2].

Molecular imaging using specific radiopharmaceuticals that target HER2 could offer a non-invasive option for better quantification and localization of HER2 overexpression and, thus, identify patients who may benefit from HER2-directed therapy. Several imaging tracers targeting HER2 have been reported, including radiolabeled mAbs, antibody fragments (Fab or F(ab)_2_), nanobodies, or affibodies [6]. One of the most well studied radiotracers for HER2 imaging is ^89^Zr-labeled trastuzumab, which allowed visualization and quantification of uptake in HER2-positive lesions for patients with metastatic BCa and other cancers in several clinical trials [7–10]. However, due to the large molecular size (150 kDa) and the intrinsically slow blood clearance of the full-length antibody, optimal detection of lesions is seen only 4–5 days after injection and accompanied by a considerable dose exposure to healthy organs, which is several-fold higher than for PET scans with ^18^F-fluorodeoxyglucose [11]. Furthermore, uptake of ^89^Zr-labeled trastuzumab has been found to be false-positive, i.e., to occur in the absence of clinically relevant HER2 expression, in a significant fraction of patients [8].

**Fig. 1:**
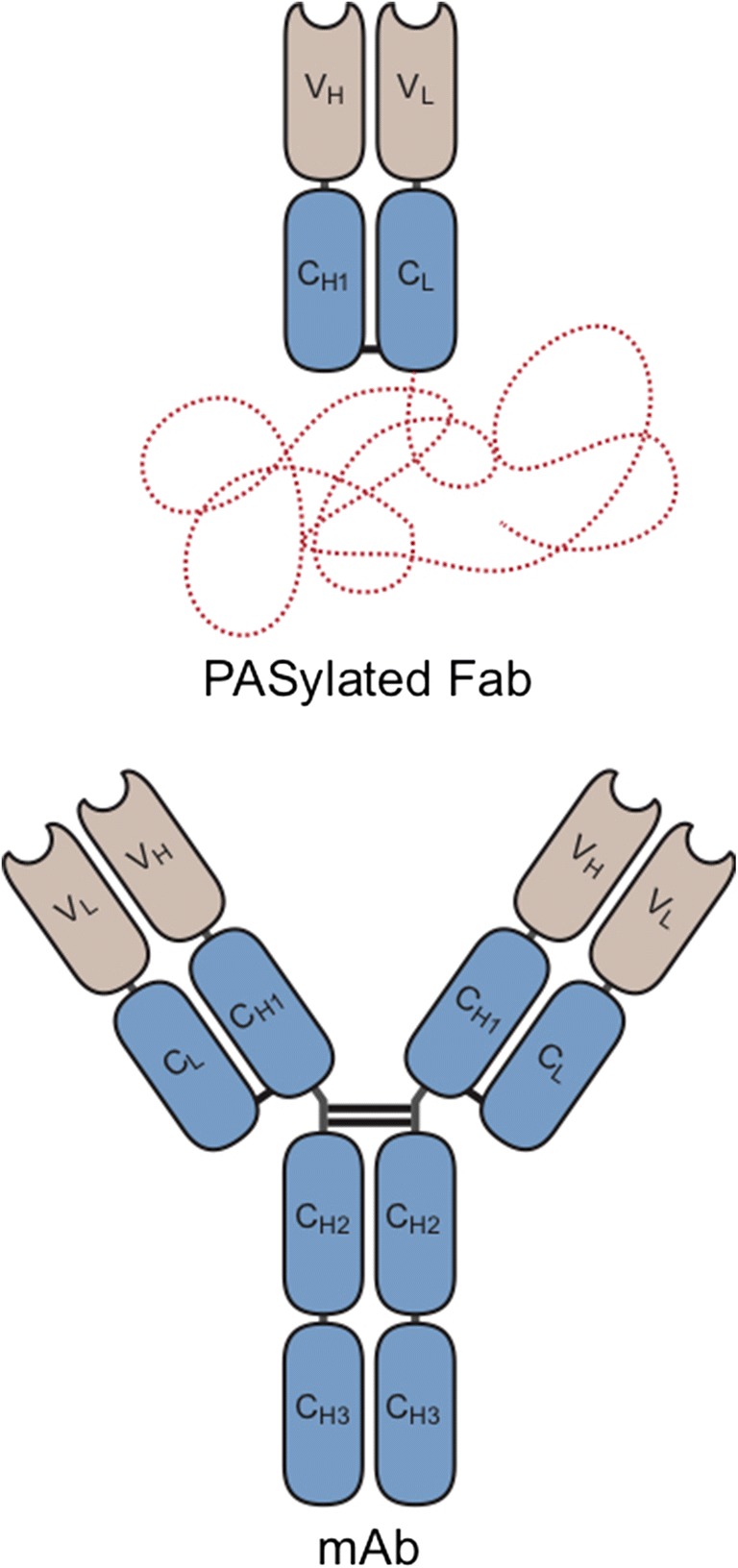
Structure of a PASylated Fab fragment in comparison with a full-size antibody

Generally, Fab fragments (48 kDa) offer rapid clearance and thereby better tumor contrast at early imaging time points. However, the fast elimination from the blood stream can also limit tumor uptake of such antibody fragments. In a preclinical study, we systematically examined the impact of plasma half-life of modified Fab fragments in a HER2-positive breast cancer model [12]. Tailoring of plasma half-life was conveniently achieved using the PASylation technology [12–14]. To this end, conformationally disordered 100–600 residue chains consisting of Pro, Ala, and Ser (PAS_100–600_) were genetically fused to the C-terminus of the light chain of the trastuzumab Fab (Fig. 1) and compared with the unmodified Fab in positron emission tomography (PET) and biodistribution experiments. In this preceding study, the radiotracer ^89^Zr∙Df-HER2-Fab-PAS_200_ revealed an optimal PET imaging contrast 24 h post injection (p.i.) in mice and, thus, appeared promising for clinical translation [15]. Here we report the first in-human PET imaging with ^89^Zr∙Df-HER2-Fab-PAS_200_ of a HER2-positive patient suffering from metastatic BCa.

## Materials and Methods

### Production of HER2-Fab-PAS_200_

The HER2-Fab-PAS_200_ [12] was produced by bench top fermentation in *E. coli* as previously described [13]. Bacterial harvest and preparation of the periplasmic extract were performed under sterile conditions by crossflow filtration in a closed system, followed by chromatographic purification to homogeneity. Conjugation of p-SCN-Bn-Deferoxamine (Df; Macrocyclics, Plano, TX) was carried out according to a published protocol [15] following good manufacturing practice guidelines. On average, 2–3 Df chelates were coupled per Fab molecule as assessed by ESI-TOF mass spectrometry.

### ^89^Zr-Labeling, Formulation, and Quality Control

Radiolabeling of Df-HER2-Fab-PAS_200_ was performed according to a published procedure [15] using ^89^Zr as supplied by Perkin Elmer (Boston, MA). Briefly, 93 MBq of ^89^Zr in oxalic acid were neutralized with Na_2_CO_3_ and incubated with 260 μg of the purified Df-HER2-Fab-PAS_200_ in HEPES/NaOH buffer (pH 7.0) for 60 min at room temperature, followed by gel filtration on a PD-10 column (GE Healthcare, Munich, Germany). Radiolabeling efficiency was 92.4%, and the radiochemical purity was > 95%, as determined by instant thin-layer chromatography (TLC). Two milliliters of the isolated ^89^Zr∙Df-Her2-Fab-PAS_200_ was diluted with 9 ml sterile 0.9% saline and sterilized by filtration through a 0.2-μm Millex LG syringe filter (Merck Millipore, Darmstadt, Germany) under aseptic conditions (with only negligible amounts of radioactivity accumulating in the filter). The amount of protein was quantified by Bradford assay (Bio-Rad Laboratories, CA) using a dilution series of the unlabeled Df-HER2-Fab-PAS_200_ preparation as reference. As a further quality control, a radio-HPLC of a sample was performed, which revealed a single peak at the expected retention time. The final product (9.6 μg/ml) was documented to be sterile and free of particles at pH 7.0, and the bacterial endotoxin content was < 0.5 EU/ml.

For the toxicity study, Df-HER2-Fab-PAS_200_ was charged with non-radioactive zirconium (^nat^Zr) using the same protocol as for the radioisotope. The product was analyzed by ESI-TOF mass spectrometry, revealing successful complexation of 1–3 ^nat^Zr ions per protein molecule.

### Single-Dose Toxicity Study

To obtain information on the general toxicity of the PASylated Fab fragment, we performed a single-dose toxicity study in female CD1-*Foxn1*^*nu*^ mice (7 months age, average weight 38.9 ± 5 g). Based on our preclinical findings [12], a maximum dose of 100 μg injected protein (“microdose”) was assessed as a starting point for the first clinical application of ^89^Zr∙Df-HER2-Fab-PAS_200_, corresponding to 1.4 μg/kg body weight for a 70-kg patient. Application of the same total protein amount to these mice was equal to a > 1000-fold dose, in line with the “ICH guideline M3(R2) on non-clinical safety studies for the conduct of human clinical trials and marketing authorization for pharmaceuticals.” Therefore, two groups of mice (*n* = 11) were injected once intravenously with 100 μg of Df-HER2-Fab-PAS_200_ charged with ^nat^Zr. The first group was sacrificed 24 h, and the second group was sacrificed 14 days after treatment with ^nat^Zr∙Df-Her2-Fab-PAS_200_. Six mice (three per group) treated with saline served as reference. All tissues and organs were examined histologically by two veterinary pathologists, and findings were reported according to the INHAND criteria of the Society of Toxicologic Pathology (STP) in line with the most recent recommendations. Hematology, clinical chemistry, and urinalysis as well as analyses of organs and blood samples were performed as described elsewhere [16]. The animal experiments were approved by local authorities (General Administration of Upper Bavaria; license 55.2-1-54-2532-46-12) and in compliance with regulatory and institutional guidelines.

### Patient

**Fig. 2:**
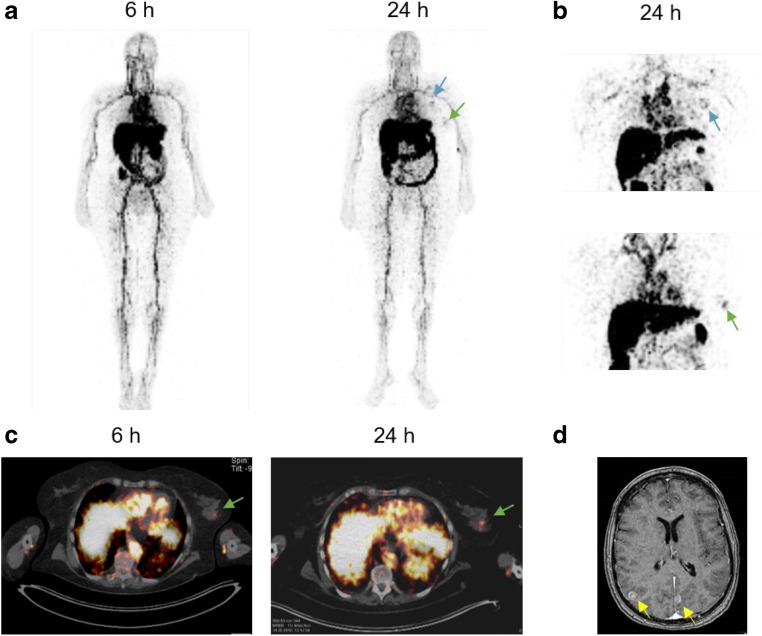
Biodistribution and lesion targeting of ^89^Zr∙Df-HER2-Fab-PAS_200_ in a mBCa patient. **a** Whole-body MIP images of ^89^Zr∙Df-HER2-Fab-PAS_200_. **b** Magnification of ^89^Zr∙Df-HER2-Fab-PAS_200_ accumulation in axillary lymph node metastases (blue arrow) and in the presumed primary tumor (green arrow) 24 h after injection. **c** Overlay of PET/CT images in the region of the presumed primary tumor in the left breast. **d** MRT scan of multiple brain metastases (yellow arrows)

^89^Zr∙Df-HER2-Fab-PAS_200_ imaging was offered to support individual therapy planning and to identify the primary tumor under the German Pharmaceuticals Act (“Arzneimittelgesetz”, AMG), Sect. 13.2b, and with notification of the General Administration of Upper Bavaria. The patient was a 67-year-old woman with newly diagnosed HER2-positive metastatic BCa. Metastatic BCa had been proven by biopsy of an enlarged axillary lymph node, but no definitive abnormal findings were seen in both breasts on mammography. On immunohistochemistry, the tumor tissue in the axillary lymph node was positive for HER2 (score 3 +). An MRI scan of the brain showed multiple enhancing lesions, consistent with metastases (Fig. 2). Thus, the tumor stage was cTx pN1 cM1. The patient was treated with whole brain radiation therapy in combination with dexamethasone (4 mg per day) prior to the ^89^Zr∙Df-HER2-Fab-PAS_200_ PET/CT scans.

### ^89^Zr∙Df-HER2-Fab-PAS_200_ Administration and PET Imaging

The radiolabeled Fab fragment (37 MBq, 70 μg) was administered intravenously over 1–2 min without premedication. The patient was monitored for at least 1 h after injection for any reactions or adverse events. During this period, the documentation of blood pressure and pulse did not show any relevant change.

### Image Acquisition and Interpretation

Image acquisition was performed using a Biograph 128 PET/CT scanner (Siemens Molecular Imaging, Knoxville, TN). Whole-body PET/CT was conducted 6 and 24 h p.i. with 13 bed positions (5 min upper body, 2.5 min lower extremities). Images of the thorax and epigastrium were acquired 45 h p.i. with 2 bed positions. For quantitative evaluation, mean standardized uptake values (SUV) were calculated from attenuation corrected image data using the average intensity of the respective tissue as listed in Table 1.

**Table 1:** Mean standardized uptake values (SUV) of ^89^Zr∙Df-HER2-Fab-PAS_200_ at different time points

Organ	6 h p.i.	24 h p.i.	45 h p.i.
Blood pool	7	4.5	2.5
Muscle	0.5	0.6	0.02
Bone	0.8	0.6	1.1
Liver	10.4	8.3	7.9
Kidney	10.6	19.0	20.0
Spleen	3.7	6.2	3.8
Lymph node filiae			
SUV_max_	3.1	5.4	4.8
SUV_mean_	0.6	0.7	0.7
Primarius			
SUV_max_	2.8	4.2	3.8
SUV_mean_	0.4	0.7	0.6

## Results

### Single-Dose Toxicity Study

Non-toxicity of Df-HER2-Fab-PAS_200_ charged with ^nat^Zr was substantiated in female mice prior to human application at > 1000-fold the clinical dose (on a mg/kg basis). No relevant histopathological findings for the main organs were detected after 24 h up to 14 days and ^nat^Zr∙Df-HER2-Fab-PAS_200_ was well tolerated.

### Biodistribution and Normal Organ Uptake

The mBCa patient underwent whole-body PET/CT imaging 6 and 24 h post injection (p.i.) with ^89^Zr∙Df-HER2-Fab-PAS_200_ (70 μg, 37 MBq). Additionally, PET images of the upper body were acquired 45 h p.i. Images showed a biodistribution typical for protein tracers, characterized by a prominent blood pool 6 h p.i., which decreased over time (Fig. 2 and Table 1). Increased uptake in the liver and kidneys was observed 24 h p.i., which—in case of the liver—decreased thereafter. Activity in the gastrointestinal tract was also prominent 24 h and 45 h p.i., suggesting hepatobiliary clearance. No significant activity was seen in the bladder, and no significant urinary excretion was noted. The ^89^Zr∙Df-HER2-Fab-PAS_200_ uptake in other non-tumor tissues (i.e., lung, muscle, bone) was low.

### Lesion Targeting

Lesions were detectable as early as 24 h p.i. of ^89^Zr∙Df-HER2-Fab-PAS_200_ (Fig. 2). The cranial lymph node metastasis in the left axillary, earlier diagnosed by CT scan, was clearly visualized by PET imaging with this novel tracer (maximum standardized uptake value (SUV_max_) = 5.4). Notably, a single lesion was also detectable in an area of dense parenchyma in the left breast, with an SUV_max_ of 4.2 at 24 h p.i., which was indicative of the putative primary tumor that had remained elusive at this time.

Of note, no accumulation of the protein tracer was visible in the known brain metastases. Furthermore, the second axillar lymph node revealed a prominent area of central necrosis with no uptake of ^89^Zr∙Df-HER2-Fab-PAS_200_.

## Discussion

Molecular imaging should help to guide physicians to tailor individual treatment of patients and to monitor the therapeutic response. To this end, biodistribution and lesion targeting of ^89^Zr∙Df-HER2-Fab-PAS_200_ was evaluated in a patient with HER2-positive mBCa. Biodistribution of ^89^Zr∙Df-HER2-Fab-PAS_200_ was in accordance with expectation from preclinical studies in mice [12, 15]. Due to the genetic fusion of the Fab with the conformationally disordered PAS_200_ polypeptide and the resulting increased hydrodynamic volume, with an enlarged apparent MW ≈ 165 kDa, ^89^Zr∙Df-HER2-Fab-PAS_200_ showed moderately prolonged blood circulation compared with the unmodified Fab [12]. Similarly, a delayed blood clearance of ^89^Zr∙Df-HER2-Fab-PAS_200_ was observed in the patient (Fig. 2; cf. time points 6 and 24 h p.i.), confirming that PASylation is also effective in humans.

In the preclinical evaluation of ^89^Zr∙Df-HER2-Fab-PAS_200_, high tumor-to-background ratios were observed 24 h p.i. (tumor-to-blood, 3.4; tumor-to-muscle, 20) with even higher values at 48 h p.i. [15]. In the patient, blood pool activity was prominent 6 h p.i. and still seen at 24 and 45 h p.i. On the other hand, signals for the lesions became more evident 24 h p.i. compared with 6 h p.i., indicating ongoing accumulation in the tumor tissue. This is in agreement with the finding from our preceding preclinical study that a moderately prolonged plasma half-life can improve tumor uptake of a Fab-size protein tracer [12]. Furthermore, our present observations indicate a longer plasma half-life of the PASylated Fab in humans than in mice. Indeed, slower clearance rates of therapeutic antibodies in humans compared with mice have been reported, in line with the rules of allometric scaling [17]. An ^111^In-labeled Fab fragment of trastuzumab was previously investigated in a phase I trial of intraoperative detection of tumor margins in patients with HER2-positive carcinoma, revealing a terminal plasma half-life of 20.7 h [18]. However, it was not feasible to reliably detect the margins of disease in those patients due to the low uptake of the ^111^In∙DTPA-trastuzumab Fab in the tumor.

In contrast to the signal for the presumed primary tumor and the metastasis in the axillary lymph node seen with ^89^Zr∙Df-HER2-Fab-PAS_200_, no accumulation of radioactivity was visible in the diagnosed brain metastases. Of note, even large molecules (e.g., T-DM1, ^89^Zr-trastuzumab) were reported to penetrate HER2-positive breast cancer brain metastases [7, 19] due to local disruption of the blood-brain barrier at these sites [20]. Possibly, in the present case, the blood-brain barrier was stabilized by the pretreatment with dexamethasone during whole brain irradiation prior to the application of ^89^Zr∙Df-HER2-Fab-PAS_200_ [21].

## Conclusions

Based on these first results, ^89^Zr∙Df-HER2-Fab-PAS_200_ has the potential to serve as a novel imaging agent to support individual therapy planning of HER2-positive BCa. PET imaging with ^89^Zr∙Df-HER2-Fab-PAS_200_ was feasible and tolerated well. This study indicates that PASylation technology is effective in a human patient and leads to delayed blood clearance of a radiolabeled anti-HER2 Fab fragment and sensitive tumor accumulation. However, the pharmacokinetics of ^89^Zr∙Df-HER2-Fab-PAS_200_ was longer than expected and may be further optimized, e.g., by use of a shorter PAS polypeptide or, possibly, by co-injection of trastuzumab in order to scavenge circulating shedded HER2 ectodomain and to effect more rapid clearance of immune complexes [22].
